# Inflammatory myofibroblastic tumour of the gallbladder

**DOI:** 10.1186/1477-7819-3-24

**Published:** 2005-04-29

**Authors:** Kasim A Behranwala, Peter Straker, Andrew Wan, Cyril Fisher, Jeremy N Thompson

**Affiliations:** 1 Gastrointestinal Surgery Unit, Royal Marsden NHS Trust, 203 Fulham Road, London SW3 6JJ, UK; 2 Department of Pathology, Royal Marsden NHS Trust, 203 Fulham Road, London SW3 6JJ, UK

**Keywords:** inflammatory myofibroblastic tumour, gallbladder tumour

## Abstract

**Background:**

Inflammatory myofibroblastic tumour (IMT) is a benign, nonmetastasizing proliferation of myofibroblasts with a potential for local infiltration, recurrence and persistent local growth.

**Case report:**

We report a case of a 51 year-old female, who had excision of a gallbladder tumour. Histopathology showed it to be IMT of the gallbladder.

**Conclusion:**

The approach to these tumours should be primarily surgical resection to obtain a definitive diagnosis and relieve symptoms. IMT has a potential for local infiltration, recurrence and persistent local growth.

## Introduction

Inflammatory myofibroblastic tumour (IMT) is a rare benign lesion that has been discussed in various organs and tissues. They are well recognised in lung and upper respiratory tract of children and young adults with a predilection for first and second decade. Intra-abdominal forms of the disease are reported to occur most frequently in the liver, followed by stomach, bowel, spleen, mesentery [1] and extrahepatic bile duct [2]. The clinical presentation will vary on the site involved. We report a case of IMT of the gallbladder, which has not been previously described. A case of inflammatory pseudotumour of the gallbladder and bile ducts with synchronous lesion in the lung, has been described, which subsided on high-dose prednisolone therapy [3]. Another case of chronic cholecystitis with features of xanthogranulomatous inflammation due to the presence of a prominent inflammatory infiltrate composed of plasma cells, lymphocytes, macrophages, foamy histiocytes and huge fibroblastic and myofibroblastic proliferation was described by Corsi A et al [4].

## Case Report

**Figure 1:**
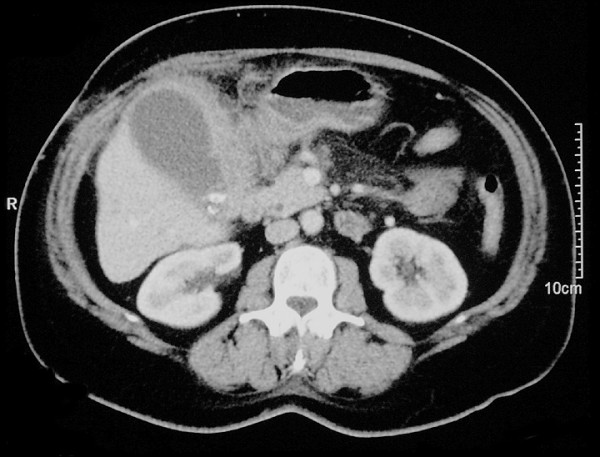
CT scan showing the gallbladder inflammatory mass

A 51-year-old female presented with history of acute right upper abdominal pain, localised abdominal signs and raised inflammatory markers. Ultrasound scanning suggested acute cholecystitis. The patient was explored initially by laparoscopy but converted to an open operation at the referring hospital. An irresectable mass, which was thought to be an advanced gallbladder carcinoma, was found. Several needle biopsies were taken from the tumour but the gallbladder was not excised. Histology of the biopsies showed features of IMT of the gallbladder. She developed obstructive jaundice postoperatively, ERCP showed a distal bile duct stricture which was stented. CT scan showed a mass lesion in the gallbladder area with direct involvement of the liver and no metastatic disease elsewhere (Figure 1). In view of the histological diagnosis the patient underwent re-laparotomy through an extended subcostal incision. Operative findings included a tense, distended gallbladder containing stones, debris and pus with a segment of transverse colon densely adherent to the mass. An en-bloc cholecystectomy and limited transverse colectomy with primary anastomosis was performed. The mass was peeled off the first and second part of duodenum, common bile duct and transverse mesocolon. Intra-operative cholangiogram via the cystic duct stump showed no biliary leakage and the dye flowed freely into the duodenum with the biliary stent in situ.

**Figure 2:**
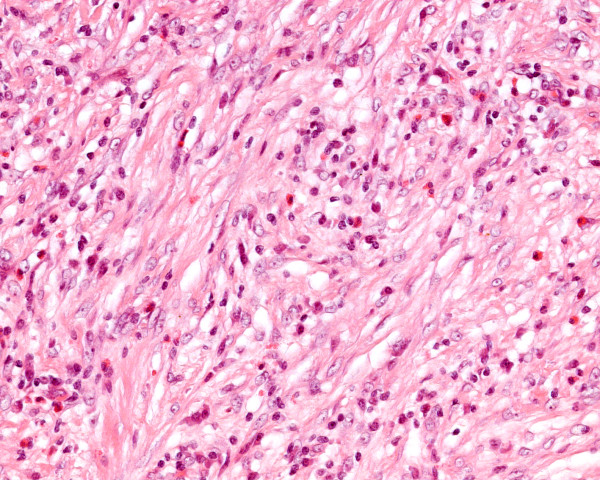
Photomicrograph showing features of inflammatory myofibroblastic tumour (Haematoxylin and Eosin × 50)

**Figure 3:**
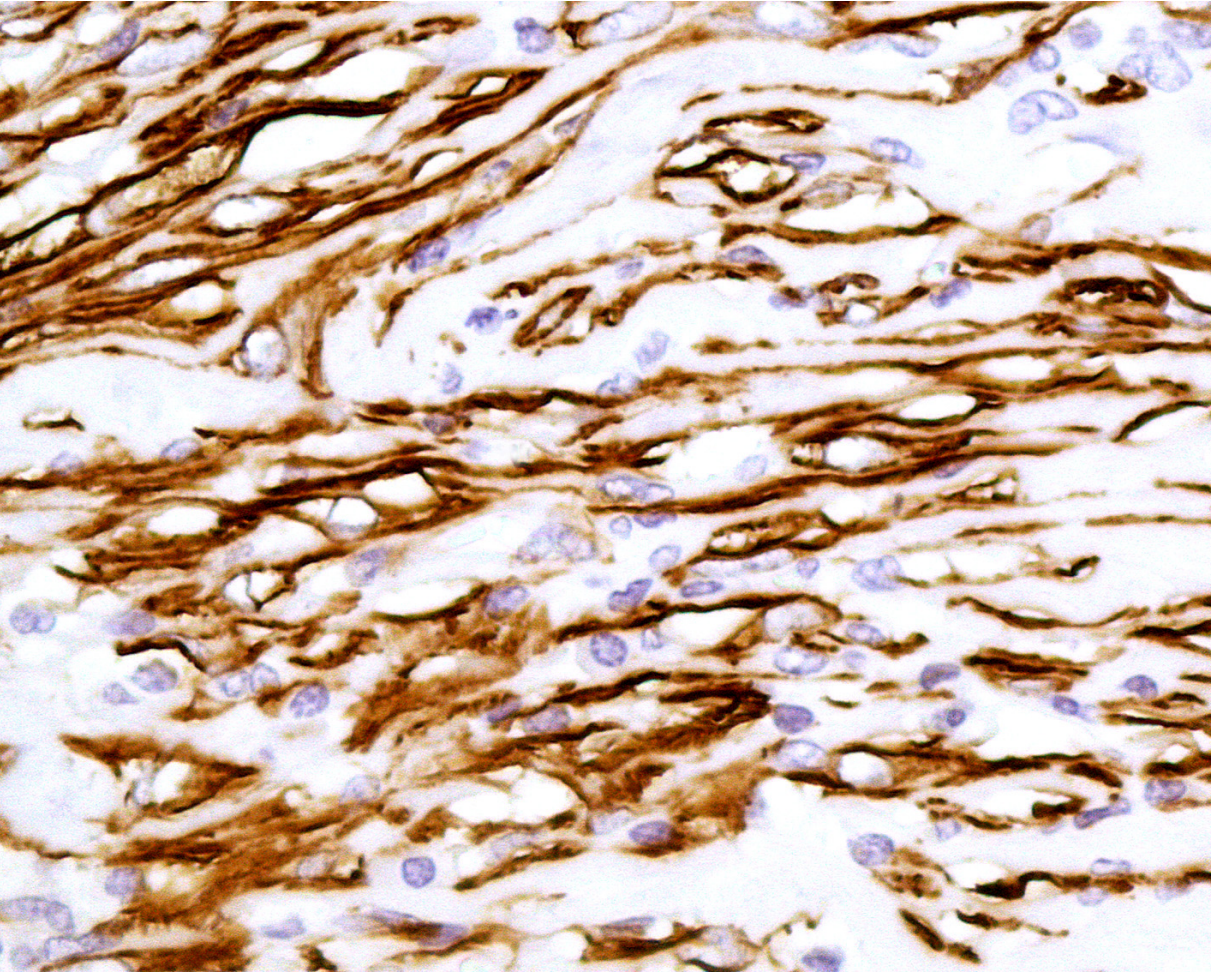
Photomicrograph showing immunohistocemical positive staining with smooth muscle actin (×100)

**Figure 4:**
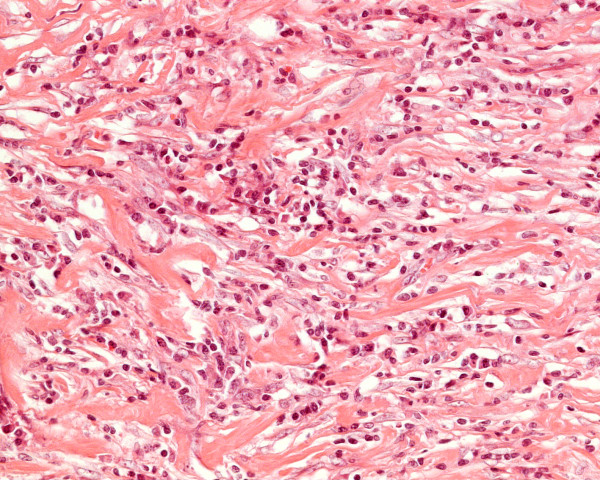
Photomicrograph showing immunohistocemical negative staining with desmin (× 100)

On pathological examination, the tumour measured 12 cms. Microscopy of the resected mass showed the gallbladder wall to be replaced by proliferative spindle myofibroblastic cells arranged in fascicles, admixed with diffuse chronic inflammatory cells including lymphocytes, plasma cells and eosinophils with lymphoid aggregates. In places hyalinised fibrous stroma was seen. No pleomorphism or vascular invasion was evident. Mitoses were inconspicuous (Figure 2). The mass was seen to grow in an infiltrating pattern with entrapment of adipocytes and extending to the muscularis propria of colon from externally. The tumour extended to the resection margin. Four lymph nodes showed reactive changes. Immunhistochemistry showed positivity for SMA (Figure 3) and calponin. ALK-1 was equivocal. However desmin, caldesmon and CAM 5.2 were negative (Figure 4).

The postoperative period was uneventful. ERCP was done at four weeks after operation with replacement of the stent because of slight stricturing of the common hepatic duct. No local recurrence was detected at six months follow-up on CT scan.

## Discussion

Inflammatory myofibroblastic tumour is a benign, non-metastasizing proliferation of myofibroblasts with potential for recurrence and persistent local growth, similar in some respect to the fibromatoses [5]. IMT is associated with constitutional symptoms and it has been variously termed plasma cell granuloma, inflammatory pseudotumour, inflammatory myofibrohistiocytic proliferation to reflect divergent views concerning its pathogenesis and level of malignancy. The attributes of a myofibroblast places it midway between a fibroblast and a smooth muscle cell and it appears capable of functional and phenotypic modulation.

Myofibroblastic tumours fall into four main groups: the family of reactive fascitis like lesions, a group of benign lesions (e.g. Mammary myofibroblastoma, intranodal myofibroblastoma, angiomyofibroblastoma and dermatomyofibroma), the locally aggressive fibromatoses (either superficial or deep) which share features of fibroblasts and myofibroblasts in varying degree and finally sarcomas showing myofibroblastic differentiation (low grade lesions – infantile myofibroblastic sarcoma, inflammatory myofibroblastic tumours, low grade myofibroblastic sarcoma, inflammatory fibrosarcoma [6] and high grade lesions – malignant fibrous histiocytoma) [7]. An aberrant or exaggerated response to tissue injury without an established cause has generally been favoured as the pathogenesis of the inflammatory pseudotumour or IMT. An immunological pathogenesis remains a possibility. Tumours with myofibroblast as the principal cell type are designated as IMT. The IMT and inflammatory fibrosarcoma appear to have many overlapping clinical and pathological features. These tumours are histogenetically related and if they are separate entities, they are differentiated more by degrees than absolutes [8]. IMT of the gastrointestinal tract is extremely rare and differ clinically, histologically and immunohistochemically from inflammatory fibroid polyps though both have a prominent inflammatory infiltrate admixed with spindle-shaped fibroblasts/ myofibroblasts set in a collagenous, fibrovascular or myxoid stroma [9].

The inflammatory myofibroblastic tumours are well circumscribed but rarely encapsulated. They usually have a homogenous consistency although areas of haemorrhage, necrosis or calcification may be found. Multicentric lesions are rare. IMT is composed of fascicles of bland myofibroblastic cells admixed with a prominent inflammatory infiltrate consisting of lymphocytes, plasma cells, macrophages and eosinophils. The lack of atypia, hyperchromasia and abnormal mitotic figures are pointers towards a benign lesion. The spindle cells stain positively for smooth muscle actin and vimentin but are negative for S100, desmin, CD100, cytokeratin, CD35 and latent membrane protein. Differential diagnosis includes calcifying fibrous pseudotumour [10,11] inflammatory fibrosarcoma, follicular dendritic cell tumour and gastrointestinal autonomic nerve tumours. Calcifying fibrous pseudotumour is a benign fibrous lesion characterised by three distinctive features: a collection of dense, hyalinized collagen interspersed with benign-appearing spindle cells, psammomatous or dystrophic calcification, and a lymphoplasmocytic inflammatory infiltrate of variable intensity [11]. Inflammatory fibrosarcoma is histogenetically related but is regarded as malignant on the basis of the high rate of local recurrence, multiple peritoneal implants, locally aggressive behaviour, distant metastases and tumour related deaths. IMT and inflammatory fibrosarcoma may be two ends of a part of a neoplastic continuum of myofibroblastic proliferation with increasing cellular atypia and aggressiveness [8]. ALK immunostaining is detected in 36% to 60% of cases in a fibrillary or granular distribution in cytoplasm or nucleus, with occasional cell and nuclear membranous accentuation [12]. The presence of chromosomal aberrations indicates that IMT is a neoplastic proliferation [13]. IMT are clonal and a proportion (30 – 40%) has reproducible cytogenetic abnormalities involving the region of the anaplastic lymphoma kinase (ALK1) gene on chromosome 2 [10]. Inflammatory myofibroblastic tumours show a wide spectrum of cellular atypia and biological behaviour with p53 and MDM2 expression. However the alterations in the p53 pathway seem not to play a major role in the pathogenesis of inflammatory myofibroblastic tumour [14].

There is no regular vascular pattern thus there is variable contrast enhancement on CT scans. On magnetic resonance imaging, hepatic IMT appears as a mass or as an area of periportal soft tissue infiltration. The mass is hypointense on T1-W and slightly hyperintense relative to surrounding liver parenchyma on T2-W image [15]. The prognosis of this tumour is generally considered to be favourable with no reports of malignancy [16]. Twenty-two cases have been reported in the pancreatic region [17]. Coffin CM et al [5] have reported 84 cases with a median age of 9 years (3 months to 46 years) occurring at various sites (abdomen, retroperitoneum or pelvis (61 cases); head and neck including upper respiratory tract (12 cases); trunk (8 cases); and extremities (3 cases)) and ranging in size from 1 to 17 cms. Excision was performed in 69 cases and clinical follow-up in 53 cases revealed 13 patients (25%) had one or more recurrences at intervals of 1–24 months. Histological confirmation is always required before diagnosis and treatment, to differentiate it from malignant tumour. Misdiagnosis has led some patients to be inappropriately treated with chemotherapy [12]. The therapeutic approach to these tumours should rely primarily on surgical resection in order to obtain a definitive diagnosis as well as to relieve symptoms. IMT has a potential for local infiltration, recurrence and persistent local growth. Local recurrence may occur many years later and thus strict follow-up after surgery is required.
